# Thermal Inactivation of *Salmonella enterica* and *Listeria monocytogenes* in Quesillo Manufactured from Raw Milk

**DOI:** 10.1155/2022/2507867

**Published:** 2022-06-30

**Authors:** Mayra Márquez-González, Luis F. Osorio, Carmen G. Velásquez-Moreno, Alvaro G. García-Lira

**Affiliations:** ^1^Food Science and Technology Department, Pan-American Agricultural School, Zamorano University, San Antonio de Oriente, Honduras; ^2^EARTH University, Puerto Limon, Costa Rica

## Abstract

Quesillo is an artisanal Honduran cheese made from raw milk. During fabrication, curd melting is considered a killing step for pathogenic bacteria. This work was aimed at determining the survival of *Salmonella enterica* and *Listeria monocytogenes* on inoculated curd packaged in plastic bags and immersed in a water bath at 48, 54, 60, 65, and 70°C for predetermined times. Survival counts of each pathogen were used to estimate *D* values by linear regression, and *z* values were estimated by the linear regression of the *D* values. *S. entericaD* values ranged from 4.5 min at 60°C to 0.80 min at 70°C (*z* = 10.7°C). For *L. monocytogenes*, *D* values ranged from 6.08 min at 60°C to 0.90 min at 70°C (*z* = 11.3°C). Validation of 7-log reduction was performed on inoculated curd heated at 65°C for 34.7 min, and recovering enrichment procedures were used for each pathogen. Neither *S. enterica* nor *L. monocytogenes* cells were recovered after the enrichment of samples. The results obtained in this study could be applied by Honduran quesillo processors to improve the safety of their products.

## 1. Introduction

The consumption of artisanal cheese is frequent among Latin American countries, especially in the Central American region. Nevertheless, the lack of Good Manufacturing Practices (GMPs) and Standard Sanitation Operating Procedures (SSOPs) during manufacturing, packaging, distribution, and marketing poses a significant health risk for consumers. In Honduras, the formal dairy industry processes only about 35% of the milk produced in the country, and the other 65% is processed raw in artisanal cheese plants which lack basic infrastructure and equipment [1, 2].

Quesillo is a pasta filata fresh type of cheese, typical of many Latin American countries, traditionally made from raw milk and has an inherited risk for pathogens. However, in the manufacturing process, the curd is acidified and then cooked and stretched at high temperatures. This process is not standardized, but it is believed to lower the bacterial count considerably and thus extending its shelf life. Some people even believe it works as pasteurization, killing all pathogenic bacteria. Quesillo is eaten fresh with no aging requirement, unlike in other regions where raw milk cheese is highly consumed [3]. Once it has been melted and stretched, quesillo is placed in plastic containers and let to cool down for a day before it is cut and placed in plastic bags for retail sale. Quesillo is white, soft, and elastic and is frequently consumed in breakfast and dinner dishes.

Cheese is among the most consumed foods in the world but also in the top ten riskiest foods regulated by the FDA [4]. *S. enterica* and *L. monocytogenes* are the most common pathogens among cheese products. Fresh cheese and Latin American-style cheeses such as queso fresco, quesillo, and others are frequently involved in foodborne listeriosis [5–7]. According to Gérard et al. [8], the prevalence of *L. monocytogenes* in fresh cheese ranges from 0.0 to 37.5%. Gaibor Orellana et al. [9] conducted a study to determine the prevalence of pathogens in raw cheese sold in marketplaces in Tegucigalpa, Honduras. They found that out of 18 samples of cheese, two tested positive for *E. coli* O157 and five exceeded the maximum allowed count for coliforms (500 CFU/g).

The objective of this study was to establish a mortality curve for *S. enterica* and *L. monocytogenes* according to the current process used in the manufacture of quesillo to later determine if the temperature and time used would render the quesillo free of these two pathogens.

## 2. Materials and Methods

### 2.1. Sample Preparation and Collection

Three batches of cheese curd were manufactured at the dairy plant at Zamorano University for further acidification and melting into quesillo. The milk used for this study was from the university dairy farm, freshly obtained (<1 h from milking), free of antibiotics, and with historically good microbiological quality. Quesillo was prepared from the acidified raw curd; it was melted and stretched to give the final product its sensorial attributes. A procedure for the manufacture of a typical artisanal Latin American quesillo is shown by Ramírez-Navas and Rodríguez de Stouvenel [10].

### 2.2. Chemical Properties of the Curd

To assess the substrate where the pathogens were studied, the chemical characteristics of the curd were determined. Fat content was determined by the Babcock method [11]; water activity was measured using the AquaLab meter (Series 3TE, Decagon Devices, Inc.); moisture content was determined by drying the sample at 105°C in an oven (Fisher Scientific, Model 750F) until constant weight. In addition, the final pH was measured using a Thermo Scientific Orion 5 potentiometer.

### 2.3. Description of Strains and Activation

*S. enterica* serotype Typhimurium (ATCC® 14028™), *S. enterica* serotype Poona (ATCC® BAA-1673™), *L. monocytogenes* (ATCC® 13932™), and *L. monocytogenes* (ATCC® 19112™) were used for this study. Strains were kept under 4°C and activated using 9 ml of Tryptic Soy Broth with yeast extract (TSB-YE, Neogen, Culture Media, MI), which were incubated at 37°C for 24 hours. A biconvex drop from each strain was streaked onto the surface of Tryptic Soy Agar (TSA, Neogen Culture Media, MI) plates, using a sterile loop to isolate colonies of the respective strains. To conclude with the activation, one single isolated colony was placed in TSB-YE to enrich the pure colonies of each of the four strains. Finally, a cocktail of the strains was prepared by mixing equal volumes of each culture.

### 2.4. Cheese Curd Inoculation

Portions of 250 grams of curd were inoculated with 2.5 ml of a solution containing all four strains of pathogens, aiming at obtaining an initial bacterial load of 7 logs. The inoculum and the curd were then homogenized by constant mixing in a sterile stainless-steel round pan with a sterile laboratory spatula for eight minutes: two minutes in a circular clockwise direction, two minutes counterclockwise, two minutes making up and down movements, and two minutes making left and right movements. Once the homogenization process was completed, 5 g portions of samples were aseptically weighed into 7 oz sterile Whirlpack™ sampling bags and evenly extended to reach 1-2 mm thick to ensure even exposure to heat treatments.

### 2.5. Thermal Treatments

Three repetitions of the study were conducted, and three different curd samples were collected for the study. Inoculated cheese curd packages were given the corresponding heat treatment by submerging the pouches in a water bath (Model WNE10, Memmert GmbH + Co.). Each sample was run in duplicate and exposed to five different heat treatments (48, 54, 60, 65, and 70°C). Samples were taken at the designed time intervals for each temperature: 10 min at 48°C, 5 min at 54°C, 2 min at 60°C, 50 s at 65°C, and 20 s at 70°C. The heating time ranged from 60 minutes at 48°C to 2.3 minutes at 70°C. The temperature and time intervals when the samples were taken out for the microbiological analysis are detailed in Table 1. A control was added by including a noninoculated curd sample with 1% of sterile water to compensate for the inoculated samples. In addition to treatment samples, a blank curd without inoculum and heat treatment and whole milk (3% fat) and whey were added to determine the initial load of *S. enterica* and *L. monocytogenes* in the raw milk and curd. The temperature was continuously monitored with a thermocouple (Model Temp 300, OAKTON Instruments) inserted at the center of two uninoculated packages. After heat treatment, the samples were cooled in an ice water bath and analyzed within 30 min.

### 2.6. Count of Survival Cells

The content of two 5 g pouches per treatment was mixed with 90 ml of buffered phosphate water. Each sample was then homogenized for one minute, using a masticator homogenizer (IUL Instruments). Further decimal dilutions were prepared and 0.1 ml or 1 ml aliquots (divided into 0.3, 0.3, and 0.4 ml) were spread plated onto the surface of TSA. After one hour at room temperature, TSA plates were overlaid with XLD agar (Neogen Culture Media, MI) and Oxford Listeria Agar (Neogen Culture Media) with natamycin (NCM 4047 Oxford supplement with natamycin, Neogen Culture Media) for *S. enterica* and *L. monocytogenes*, respectively. Plates were then incubated at 37°C for 24 hours for later reading. Colonies that met the corresponding morphological characteristics for *S. enterica* and *L. monocytogenes* were counted. Uninoculated samples (negative controls) were also plated to assure that counted colonies were the survival cells of spiked cultures.

### 2.7. *D* and *z* Value Determination

*D* values were determined for each pathogen at different temperatures, using DMFiT from ComBase© web interface [12]. Linear models for each of the temperatures were generated, as well as for each pathogen, *S. enterica* and *L. monocytogenes*. The model was built plotting time and CFU log of the surviving bacteria. The slope, model adjustment, and error were obtained from each thermal death curve. To estimate the *D* value, the reciprocal of the slope was calculated to determine the number of minutes required to reduce one log of the initial bacterial load. A graph using the five temperatures against the logarithm of the *D* value for each temperature was plotted. Linear regression procedures were used to determine the *z* value. The reciprocal slope obtained from the resulting curve was calculated to establish how many degrees Celsius were required to increase or reduce one logarithm of the bacterial load.

### 2.8. Validation of Results

A 300 g portion of freshly prepared curd was inoculated with 1% of the mixture of both pathogens aiming at obtaining an initial bacterial load of 7 logs of *S. enterica* and *L. monocytogenes*, respectively. Times for treatment were estimated from the 95% confidence intervals of *L. monocytogenes* obtained from the regression analyses for *z* value determination. A negative control was also tested.

Inoculated curd and negative control were packaged in 25 g portions. Using a water bath, each package was exposed to the temperature and time parameters for validation. One package was used to validate *D*_65_, a second package was used to validate *D*_70_, and a third package was used to validate *z* value to estimate the time required in the thermal treatment at 75°C and the control at 65°C. An untreated portion was used to confirm the initial pathogen load. The validation experiment was conducted in triplicate.

Each 25 g treated sample were then placed in 225 mL of Universal Preenrichment Broth and incubated at 37°C for 24 hours. After incubation, the samples were streaked in XLD Agar and Oxford Agar with natamycin using one biconvex drop from each one of the quesillo sample to ensure the absence of *Salmonella enterica* and *Listeria monocytogenes*, as well as the effectiveness of each treatment.

### 2.9. Statistical Analysis

All statistical procedures were analyzed using SAS (version 9.4) [13]. Heat resistance data were analyzed by a simple linear regression procedure. Estimation of predicted values and confidence intervals was determined with *α* = 0.05. The means procedure was used to estimate the mean and standard deviation of chemical analysis. Finally, analysis of variance (ANOVA) was used to determine if there were significant differences among the treatments (*α* = 0.05).

## 3. Results and Discussion

The pH of fresh curd used in the study was 5.72 ± 0.06, the fat content was 25.13 ± 0.25%, the humidity was 54.62 ± 2.78%, and the water activity was 0.980 ± 0.001. A linear decrease in *S. enterica* and *L. monocytogenes* populations at 60, 65, and 70°C was observed. When heated at temperatures of 54°C, two survival rates were observed, and no inactivation or growth of pathogens occurred at 48°C within 60 min. Linear inactivation of *S. enterica* during quesillo making resulted in *D* values ranging from 4.5 min at 60°C to 0.80 min at 70° C (Table 2). *L. monocytogenesD* values ranged from 6.08 min at 60°C to 0.90 min at 70°C (Table 3). No significant differences were found in the heat resistance of *S. enterica* and *L. monocytogenes* at all temperatures examined (*P* > 0.05).

**Figure 1:**
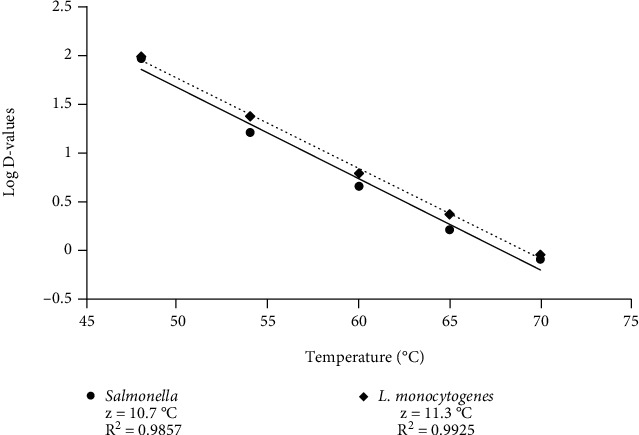
Thermal death time curve (*z* value) for *Salmonella enterica* and *Listeria monocytogenes* for a temperature range of 48 to 70°C during heating of fresh curd. Each data point represents the mean of three replicated experiments at each temperature.

The *z* values were calculated at 10.7 and 11.3°C for *S. enterica* and *L. monocytogenes*, respectively (Figure 1). The data from Tables 1 and 2 were used to predict the time required at specified temperatures to achieve a certain number log reduction on fresh curd. At 70°C, the time required to obtain 7-log reduction was in the range of 12.3 to 13.3 min for *S. enterica* and *L. monocytogenes*, respectively (Table 4). Based on the thermal death time values determined in this study, fresh curd should be heated to an internal temperature of 65°C for at least 34.7 min to achieve a 7-log reduction of *S. enterica* or *L. monocytogenes.*

### 3.1. Validation of Thermal Treatments

Thermal treatments for validation of results were conducted using the data obtained from the linear regression predictions of the *z* value. The high value with a 95% confidence interval was used to estimate a 7-log reduction (Table 4). Treatments applied to inoculated curd packages were 34.7 min at 65°C, 13.3 min at 70°C, and 5.2 min at 75°C. The initial load of inoculated fresh curd was 5.58 ± 0.476 and 6.36 ± 0.572 logs CFU/g for *S. enterica* and *L. monocytogenes*, respectively. From three inoculated samples, no *S. enterica* nor *L. monocytogenes* cells were recovered from 25 g of sample enriched with recovery media.

Some studies report that counts of *L. monocytogenes* could reach levels in the range of 10^4^ CFU/ml in naturally contaminated milk [14]. On fresh cheeses, *L. monocytogenes* counts are reported at levels ranging from 2.48 to 4.28 logs CFU/g [15]. Traditional curd stretching into quesillo making occurs manually in hot water until it reaches the proper texture.

Most of the studies of pathogen survival during curd stretching are related to mozzarella preparation [16–18]. Water temperatures used during curd stretching may range from 60 up to 85°C, and the temperature of stretched curd may range from 50 to 65°C. Temperatures evaluated in this study were aimed at covering the curd temperatures reported [19, 20]. The number of survival cells at the same temperature profiles may depend on other factors such as the kneading and pulling movements, the duration of the curd stretching step, and the ratio between curd mass and hot water. When the curd temperature reaches 78-80°C, a 5-log reduction of O157 and O26 Shiga toxin-producing *E*. *coli* has been reported [18]. Kim et al. [17] reported no inactivation of *L. monocytogene*s during curd stretching at 55°C. Inactivation of a 7-log inoculum of *L. monocytogenes* was achieved after 5 min of stretching at 66°C and 1 min at 77°C [17]. During the preparation of Turkish kashan cheese, kneading soft curd at 75°C for 5 min did not inactive inoculated *L. monocytogenes* at inoculum level of 4.71 logs CFU/ml on raw milk [21]. *S. enterica* inactivation led to 8.4- and 7.5-log reductions for *L. monocytogenes* and *S. enterica* when inoculated curd was melted and stretched with hot water at 90°C [22].

The heating process of the curd is necessary for melting the curd into quesillo. This process has been considered a possible pasteurization process. Oliszewski et al. [3] described a 3.8-log CFU/g reduction of total coliforms during traditional stretching of curd in water at 70-75°C during 20-30 s. In the same study, fecal coliform (thermotolerant) reduction was 1.4 log CFU/g. Canales et al. [23] reported a 4.7-log and 3.5-log decrease in *E. coli* and coliforms, respectively, during conventional kneading of Oaxaca cheese, a pasta filata type of Mexican cheese, at 55°C. They reported *D*_55_ values of 3.1 and 4.1 min for *E. coli* and coliforms. These results are far from our values reported with *S. enterica* and *L. monocytogenes*, which have higher resistance to thermal treatments. Although temperature and time used in heat treatments are considered a pasteurization process for fluid milk, the high-fat content of curd contributes to the increased thermal resistance of the microorganisms. Salt concentration is also another factor that can determine survival of pathogens. Ganesan et al. [24] studied the survival of bacteria in fresh mozzarella cheese and concluded that 2% salt concentration, the same as this study, was not enough to control or slow down the growth of cold- and salt-tolerant bacteria.

## 4. Conclusions

Traditional melting of curd into quesillo presents a risk of pathogen survival. Thereafter, milk pasteurization should be the first option to eliminate pathogens. Alternative melting procedures as described in this document (maintaining curd temperature at 65°C during 34.7 min) inactivate up to 5 logs CFU/g of *S. enterica* and *L. monocytogenes*. The results obtained could be applied by Honduran quesillo producers since the proposed temperature treatments may be used, assuming that the physical and chemical properties of the quesillo described in the study are the same. Any change in fat content, humidity, and salt content should also be validated. The heating of curd should not be a substitute for following hygienic practices during quesillo manufacture.

## Figures and Tables

**Table 1:** Description of temperature and time intervals for collecting samples of inoculated fresh curd during quesillo making at 48-70°C.

Temperature (°C)	Time intervals
48	0, 2, 10, 20, 30, 40, 50, and 60 min
54	0, 5, 10, 15, 20, 25, 30, and 35 min
60	0, 2, 4, 6, 8, 10, 12, and 14 min
65	0, 50, 100, 150, 200, 250, 300, and 350 s
70	0, 20, 40, 60, 80, 100, 120, and 140 s

**Table 2:** *D* values (obtained from linear regression) for a cocktail of two strains of *Salmonella enterica* inoculated in fresh curd during quesillo making at 48-70°C.

Temperature (°C)	Maximum rate	*D* value (min)	*R* ^2^	SE of fit
48	−0.0109 ± 0.0056	91.74	0.488	0.232
54	−0.0623 ± 0.0148	16.05	0.878	0.282
60	−0.2222 ± 0.0327	4.50	0.922	0.323
65	−0.6182 ± 0.1098	1.62	0.835	0.553
70	−1.2430 ± 0.1653	0.80	0.928	0.281

Maximum rate values are the mean ± standard deviation of three replicated experiments.

**Table 3:** *D* values (obtained from linear regression) for a cocktail of two strains of *Listeria monocytogenes* inoculated in fresh curd during quesillo making at 48-70°C.

Temperature (°C)	Maximum rate	*D* value (min)	*R* ^2^	SE of fit
48	−0.0104 ± 0.0048	96.15	0.706	0.155
54	−0.0427 ± 0.0089	23.42	0.792	0.263
60	−0.1644 ± 0.0215	6.08	0.936	0.210
65	−0.4314 ± 0.0850	2.32	0.870	0.336
70	−1.1173 ± 0.1074	0.90	0.867	0.352

Maximum rate values are the mean ± standard deviation of three replicated experiments.

**Table 4:** Predicted *D* values and 95% confidence intervals for *Salmonella enterica* (*z* = 10.7°C) and *Listeria monocytogenes* (*z* = 11.3°C) at different temperatures.

Microorganism	Temperature (°C)	Predicted *D* value (min)	95% confidence interval for predicted *D* value (min)		*F*_7D_ (min)
*Salmonella*	60	5.37	2.16	13.32	93.2
	61	4.33	1.74	10.77	75.4
	62	3.49	1.40	8.73	61.1
	63	2.81	1.12	7.09	49.6
	64	2.27	0.89	5.77	40.4
	65	1.83	0.71	4.71	33.0
	66	1.47	0.56	3.85	27.0
	67	1.19	0.45	3.16	22.1
	68	0.96	0.35	2.59	18.2
	69	0.77	0.28	2.13	14.9
	70	0.62	0.22	1.76	12.3

*Listeria*	60	7.19	3.86	13.40	93.8
	61	5.86	3.14	10.94	76.6
	62	4.78	2.55	8.95	62.7
	63	3.90	2.07	7.34	51.4
	64	3.18	1.67	6.02	42.2
	65	2.59	1.35	4.95	34.7
	66	2.11	1.09	4.08	28.5
	67	1.72	0.88	3.36	23.5
	68	1.40	0.71	2.78	19.4
	69	1.14	0.57	2.30	16.1
	70	0.93	0.46	1.90	13.3
	75	0.34	0.15	0.75	5.2

## Data Availability

The data used to support the findings of this study are available from the corresponding author upon request.
